# End-stage cystic fibrosis lung disease is characterised by a diverse inflammatory pattern: an immunohistochemical analysis

**DOI:** 10.1186/s12931-016-0489-2

**Published:** 2017-01-10

**Authors:** Elise J. Lammertyn, Elly Vandermeulen, Hannelore Bellon, Stephanie Everaerts, Stijn E. Verleden, Kathleen Van Den Eynde, Ken R. Bracke, Guy G. Brusselle, Pieter C. Goeminne, Erik K. Verbeken, Bart M. Vanaudenaerde, Lieven J. Dupont

**Affiliations:** 1 Laboratory of Respiratory Diseases, Department of Clinical and Experimental Medicine, University of Leuven, UZ Herestraat 49, Box 706, 3000 Leuven, Belgium; 2 Translational Cell and Tissue Research Unit, Department of Imaging and Pathology, University of Leuven, Leuven, Belgium; 3 Department of Respiratory Medicine, Ghent University Hospital, Ghent, Belgium; 4 Cystic Fibrosis Unit, Department of Respiratory Diseases, University Hospitals Leuven, Leuven, Belgium

**Keywords:** Cellular immune system, Histopathology, Lung immunopathology

## Abstract

**Background:**

Cystic fibrosis (CF) lung disease is characterised by vigorous airway inflammation eventually resulting in severe lung damage. This study aimed to describe the diversity of the inflammatory pattern in end-stage CF lungs by evaluating and quantifying which components of the innate and adaptive immunity are involved, and by assessing whether this is gender-specific.

**Methods:**

CF explant lung tissue (*n* = 20) collected at time of transplantation and control tissue (*n* = 22) was sectioned (9 μm) and stained for neutrophils, eosinophils, mast cells, dendritic cells, macrophages, CD4 T cells, cytotoxic T cells and B cells. Quantification with special attention for immune cell location was performed.

**Results:**

Neutrophils, mast cells, dendritic cells, macrophages, CD4 T and cytotoxic T cells were significantly increased in CF compared to controls and there was a disproportionate increase of neutrophils around the airways in CF. Large amounts of lymphoid follicles were found in the CF lung and they had a skewed B cell/T cell composition. Upon subdividing the CF patients into a male and female population, eosinophils, mast cells and CD4 T cells were increased specifically in CF females. In this subpopulation, lymphoid follicles had less B cells and more CD8 T cells.

**Conclusion:**

These data demonstrate a diverse inflammatory response in the CF lung, reflected by an increase of both myeloid and lymphoid immune cells. Inflammation in the CF lung appeared to be gender-specific in our population, as the significant increase of eosinophils, mast cells and CD4 T cells was especially notable in the female subpopulation.

**Electronic supplementary material:**

The online version of this article (doi:10.1186/s12931-016-0489-2) contains supplementary material, which is available to authorized users.

## Background

Cystic fibrosis (CF) is the most prevalent autosomal recessive disorder in the Caucasian population and is caused by mutations in the cystic fibrosis transmembrane conductance regulator (CFTR) gene [1, 2]. Loss of CFTR-mediated chloride and bicarbonate transport leads to dehydration of the airway surface fluid layer and impaired mucociliary clearance. As a result, desiccated secretions obstruct the airways and prevent bacterial elimination, establishing chronic infection, commonly with *Pseudomonas aeruginosa*, and leading to bronchiectasis [3, 4]. These events are followed by vigorous airway inflammation and may eventually result in severe lung damage, the principal cause of death or reason for transplantation in these patients [4–6].

Neutrophil-dominated airway inflammation of the CF lung environment is a well-established concept: bacterial pathogens interact with epithelial cells and alveolar macrophages, which are part of the lung’s innate cellular surveillance system, causing them to release pro-inflammatory mediators leading to excessive recruitment of neutrophils into the bronchoalveolar compartments of the lung [7, 8]. When chronic infection is established, dendritic cells and T cells also become activated, the latter releasing even more cytokines contributing to the excessive and perpetual recruitment of neutrophils. This immune response is unable to resolve the infection and massive amounts of oxidants and proteases are released [7, 8]. The inflammatory cellular pattern of CF lung disease is presumably diverse, given the many different mediators present, but remains insufficiently studied. Besides the key players listed above, data on other immune cells within the CF lung such as eosinophils, mast cells, cytotoxic T cells and B cells, are scarce [9–12]. A diverse inflammatory pattern in CF may also be anticipated based on the genotypic variation and the broad range of bacteria and fungi attacking the lungs, which may result in a pathogen-specific CD4 T cell response [7].

Another phenomenon indicative of the diversity of CF lung disease is the CF ‘gender gap’. There seems to be a discrepancy between the median survival of male and female CF patients. This CF ‘gender gap’ was first described in the 1990’s [13]. A large retrospective cohort analysis of the US CF registry, comprising over 20 000 patients between 1988 and 1992, showed that females had a 60% greater chance of dying compared to males, and this effect remained significant even after adjusting for lung function, pancreatic insufficiency and age at diagnosis [14]. Harness-Brumley *et al.* demonstrated that in 2014, CF females still have a decreased median life expectancy (36.0 years versus 38.7 years in males) and that female gender is a risk factor for death [15]. Morbidity is also increased in female CF patients, with a significantly higher use of intravenous (I.V.) antibiotics, macrolides and steroids, and an increased number of days spent in the hospital [16].

In this study, we aimed to describe the diversity of the inflammatory pattern in the CF lung by quantifying and localising the different immune cells within end-stage CF lungs. Furthermore, we wanted to investigate whether there is a different inflammatory signature in male versus female CF patients.

## Methods

### Study material

CF explant lung tissue was collected at the moment of lung transplantation (LTx) (procedures performed between 2000 and 2012). Control lung tissue was obtained from two different patient populations: firstly, patients who had no underlying lung disease and had a non-respiratory cause of death (abscess aorta, rectal adenocarcinoma, chronic kidney insufficiency, acute liver failure, sepsis, pancreatitis, ALS, hemoperitoneum) and underwent autopsy, and secondly, patients with a non-metastasized lung tumour. In this case, tissue was taken as far away from the tumour as possible. For the former group, lung function tests were not available, and for the latter, patients were only included if they had a lung function within normal limits. The use of lung tissue for scientific research was approved by the local ethics committee (S52174) and the biobankboard (S51577).

Patient data were collected via the electronic patient files or via the referring centre. Details on the collected data are mentioned in Additional file 1.

### Immunohistochemistry

Nine μm thick sections (mean surface area: 322 mm^2^) were prepared from formalin-fixed paraffin-embedded tissue from each of the subjects and stained for CD4 T cells, cytotoxic T cells (CD8), dendritic cells (CD1a and CD207), eosinophils (EG-2), mast cells (tryptase), neutrophils (MPO) and macrophages (CD163). Additional details and an overview of all used primary and secondary antibodies together with the appropriate chromogen are provided in Additional file 1.

### Image analysis

Images of tissue sections were recorded with a BX61 light microscope (Olympus, Aartselaar, Belgium). All myeloid cells (dendritic cells, neutrophils, macrophages and mast cells) were counted in 10 randomly selected high-power fields (HPF) per three compartments (airway, parenchyma and perivascular). Parenchyma was defined as the absence of airways and blood vessels. Cell countings in the perivascular compartment did not include cells lying inside the lumen of the vessel. In the case of a HPF including both an airway and an accompanying blood vessel, only the cells in the immediate proximity of the airway were counted. All cell types were captured with a 200× magnification. Cell type counts were expressed as cells per HPF for the three compartments separately and also in total, which was an average of the counts in the different compartments. Staining reliability and quality was verified by an experienced pathologist (EKV) before analysis. To assess counting reliability, inter-and intra-observer variability was calculated by means of a Spearman’s rank correlation coefficient. Myeloid cell counts were repeated by the first author (EJL) and the second author (EV) in eight subjects (four randomly chosen controls and four CF patients) (Additional file 1: Table S2).

For lymphoid B (CD20) and T (CD4, CD8) cells, quantification was different as it was performed by counting all scattered cells and follicles (*i.e.* cells aggregated as lymphoid tissue) visible on the section and normalizing the result over the total area of the section. This resulted in the number of scattered cells and follicles being expressed as cells or follicles per mm^2^ area unit. This method was used because of the inhomogeneous spread of lymphoid cells (*i.e.* presence of follicles). As such, classification of the scattered cells under one of the three compartments was not possible. Next, the percentage of positive B and T cells within the follicle was estimated. For each staining (CD20-CD4-CD8), we allocated a percentage (in steps of 10%) of positivity to each individual follicle (therefore, each follicle was included in the analysis) which we used to study differences in composition. Also, the size and localization of the follicles (airways, parenchyma, and perivascular) was noted. More details on follicle analysis and exact numbers of follicles counted are provided in Additional file 1.

### Statistical analysis

GraphPad Prism 4.0 Software (San Diego, CA, USA) was used for univariate statistical analysis. Results are expressed in numbers (percentage), as mean ± SEM or as median (IQR). When appropriate, differences in categorical variables were determined using a chi square test. Differences in continuous variables between two groups (control subjects and CF patients) were tested using a Mann-Whitney U test, and for comparison between three groups (compartments and control subjects vs. male CF patients vs. female CF patients), a Kruskal-Wallis 1-way analysis of variance (ANOVA) in combination with a Dunn’s post hoc test was used. A *p*-value <0.05 was considered significant. Intra-observer and inter-observer reliability was evaluated by the calculation of Spearman’s rank correlation coefficients.

## Results

### Patient characteristics

CF patients (*n* = 20) were significantly younger than control patients (*n* = 22) (*p* < 0.0001). There was no significant difference in gender (*p* = 0.77). There were no significant differences in patient characteristics between male and female CF patients, although a trend towards significantly lower age and higher FEV_1_ was seen in females (Table 1).

**Table 1:** Demographic characteristics of control subjects and CF patients, with a distinction between males and females

Characteristics of control subjects and CF patients			
	*Control subjects*	*CF patients*	*p-value*
*Number of patients (n)*	22	20	
*Female gender (n)*	10 (45%)	10 (50%)	0.77
*Mean age (±SEM) (years)*	61.0 (±2.7)	29.5 (±3.5)	<0.0001***
Characteristics of male and female CF patients			
	*Male*	*Female*	*p-value*
*Number of patients (n) (%)*	10 (50%)	10 (50%)	1.00
*Mean age (±SEM) (years)*	31.6 (±3.5)	27.3 (±6.1)	0.052
*Mean FEV*_*1*_ *(±SEM) (% predicted)*	25.6 (±2.3)	30.6 (±1.5)	0.052
*CFTR genotype (n) (%)*			
*ΔF508/ΔF508*	4 (40%)	6 (60%)	0.30
*ΔF508/other*	4 (40%)	4 (40%)	
*other/other*	2 (20%)	0 (0%)	
*Nasal polyposis (n) (%)*	4 (40%)	2 (20%)	0.33
*High urgency listing prior to LTx (n) (%)*	1 (10%)	4 (40%)	0.12
*Time on waiting list prior to LTx (±SEM) (days)*	270.6 (±147.2)	139.6 (±61.8)	0.25
*CRP (±SEM) (mg/L)*	50.5 (±31.0)	28.0 (±8.4)	0.80
*Differential eosinophil count on total WBC (±SEM) (%)*	2.2 (±0.9)	2.1 (±0.8)	0.97
*Total IgE (±SEM) (kU/L)*	89.2 (±38.0)	55.8 (±14.3)	0.91
*Colonization with P. aeruginosa (n) (%)*	8 (80%)	6 (60%)	0.33
*Fungi cultured from explant lung material (n) (%)*	7 (70%)	6 (60%)	0.64
*Use of inhaled corticosteroids (n) (%)*	7 (70%)	7 (70%)	1.00
*Use of oral corticosteroids (n) (%)*	2 (20%)	1 (10%)	0.39
*Time to last pre-LTx I.V. AB therapy (± SEM) (days)*	48.5 (±19.1)	77.9 (±61.0)	0.48

*Abbreviations*: *FEV*_*1*_ Forced Expiratory Volume in 1 s, *CRP* C-reactive protein, *WBC* White blood cells, *I.V.* Intravenous, *AB* Antibiotic. Significant differences between CF patients and control subjects are indicated with *, with * = *p* < 0.05, ** = *p* < 0.01 and *** = *p* < 0.001

data < 0.05 are captured in bold

### Myeloid cell quantification and localization in end-stage CF lung tissue

**Fig. 1:**
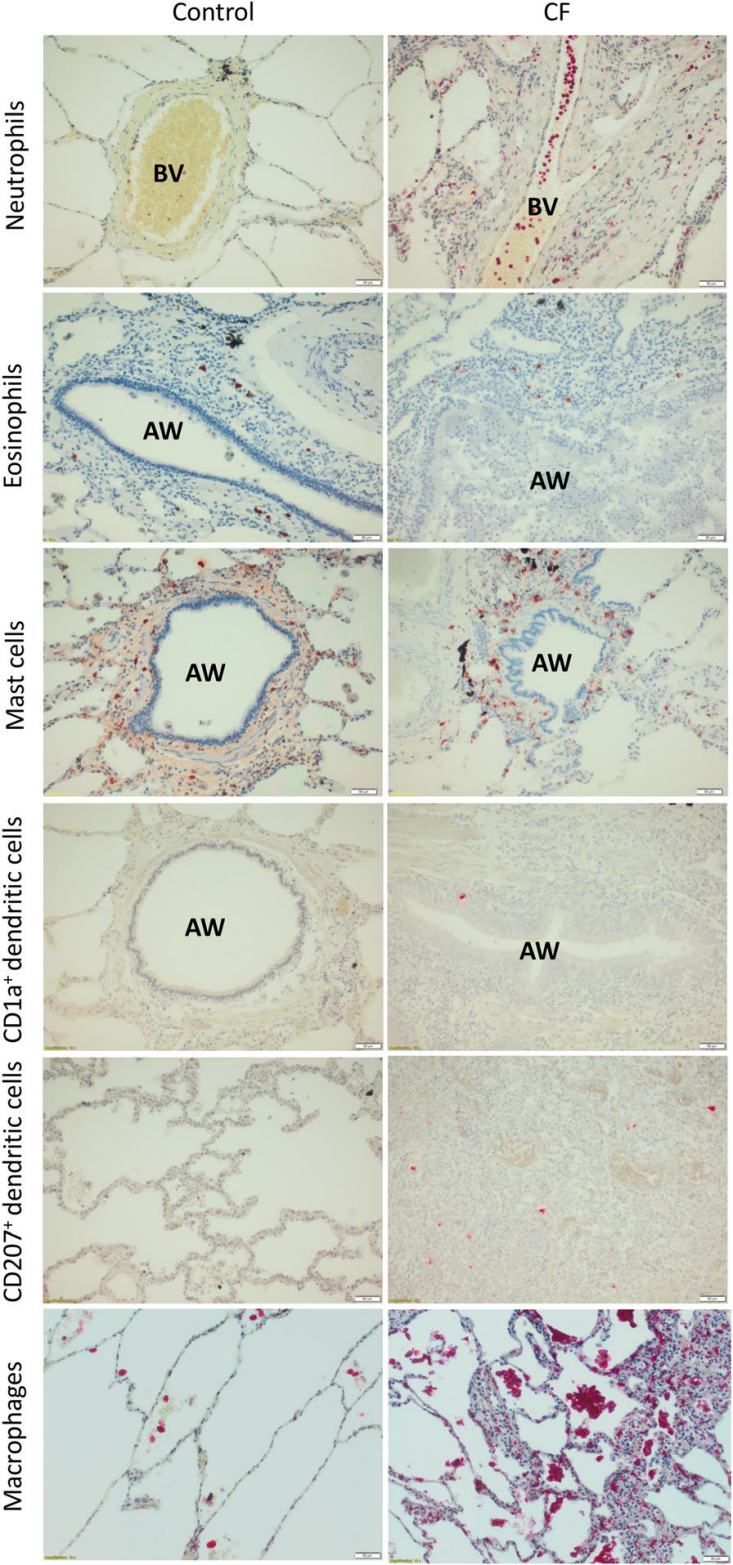
Histological sections of formalin-fixed paraffin-embedded human lung tissue of control subjects and CF patients. Sections were stained for neutrophils (MPO), eosinophils (EG-2), mast cells (tryptase), dendritic cells (CD1a and CD207) and macrophages (CD163). Scale bar = 50 μm. Abbreviations: AW = airway, BV = blood vessel

Neutrophil counts were significantly increased in CF tissue compared with controls (*p* = 0.0024), which was due to an increased presence of neutrophils around the CF airways (*p* < 0.0001) (Table 2 and Fig. 1). In CF patients, neutrophils were disproportionally more prevalent around the airways as they were found more than twice as much in this compartment compared with parenchyma (*p* < 0.01) or perivascular (*p* < 0.001) (Additional file 1: Table S4).

**Table 2:** Quantification of myeloid cell types in controls and CF overall and for the three compartments

Myeloid cell quantification and localization (expressed as cells/HPF)			
	*Controls*	*CF*	*p-value*
*Neutrophils (MPO)*	17.4 (7.1–30.5)	33.7 (25.4–47.8)	0.0024**
*Airway*	15.1 (6.3–28.7)	67.5 (43.5–81.9)	<0.0001***
*Parenchyma*	19.7 (7.1–40.6)	28.9 (15.3–36.6)	0.18
*Perivascular*	9.7 (3.5–19.4)	15.2 (9.2–18.9)	0.18
*Eosinophils (EG-2)*	1.0 (0.4–2.9)	2.3 (0.3–5.0)	0.82
*Airway*	1.7 (0.6–5.5)	2.0 (0.5–7.7)	0.10
*Parenchyma*	1.1 (0.3–2.7)	1.1 (0.1–3.6)	0.63
*Perivascular*	0.6 (0.1–1.7)	0.4 (0.0–2.0)	0.61
*Mast cell (tryptase)*	10.5 (8.8–14.4)	17.6 (12.5–24.7)	0.018*
*Airway*	18.8 (15.3–31.2)	25.2 (19.9–36.6)	0.21
*Parenchyma*	7.8 (5.0–9.2)	15.0 (8.8–21.3)	0.0024**
*Perivascular*	9.3 (6.9–11.1)	12.5 (9.9–15.8)	0.0082**
*Dendritic cells (CD1a)*	0.8 (0.4–1.5)	3.0 (2.1–4.2)	<0.0001***
*Airway*	2.3 (0.8–3.6)	4.7 (3.0–6.3)	0.0013**
*Parenchyma*	0.2 (0.0–0.5)	2.2 (1.0–3.8)	<0.0001***
*Perivascular*	0.7 (0.1–2.0)	1.6 (0.9–3.5)	0.012*
*Dendritic cells (CD207)*	1.0 (0.3–1.8)	4.6 (3.7–7.6)	<0.0001***
*Airway*	3.0 (1.6–4.7)	9.6 (7.6–13.2)	<0.0001***
*Parenchyma*	0.0 (0.0–0.2)	2.7 (1.1–5.9)	<0.0001***
*Perivascular*	0.1 (0.0–0.4)	1.2 (0.5–4.0)	0.0001***
*Macrophages (CD163)*	18.7 (10.3–25.1)	32.0 (23.0–39.8)	0.0021**
*Airway*	15.5 (8.7–26.6)	33.8 (18.4–40.4)	0.0049**
*Parenchyma*	21.3 (10.6–26.8)	35.0 (21.9–46.8)	0.011*
*Perivascular*	16.9 (11.0–21.0)	22.4 (14.1–31.6)	0.064

Quantification of the myeloid cell types in control subjects and CF patients overall and for the three compartments (airway, parenchyma and perivascular) separately. The *p*-values in the right-hand column are the result of Mann-Whitney U testing. Significant differences between CF patients and control subjects are indicated with *, with * = *p* < 0.05, ** = *p* < 0.01 and *** = *p* < 0.001

data < 0.05 are captured in bold

There was no difference in the number of eosinophils between control and CF tissue, regardless whether we considered the whole sample or analysed per compartment (Table 2, Fig. 1).

Mast cell counts were significantly increased in CF tissue compared with controls (*p* = 0.018) due to higher numbers of mast cells in the parenchyma (*p* = 0.0024) and perivascular (*p* = 0.0082) (Table 2, Fig. 1). Both in CF and control patients, mast cells occurred proportionally more around airways compared with parenchyma (*p* < 0.05 and *p* < 0.001 respectively) or perivascular (*p* < 0.01 and *p* < 0.001, respectively) (Additional file 1: Table S4). In the CF tissue, we were able to identify 19 airways showing constrictive bronchiolitis (representative image: Additional file 1: Figure S1). Mast cells were counted and compared to mast cell counts around 19 representative bronchiectatic airways, and there were significantly more mast cells around airways showing constrictive bronchiolitis (48 cells/HPF, IQR 36–57) than around bronchiectatic airways (28 cells/HPF, IQR 15–45) (*p* = 0.035).

CD1a (immature myeloid) dendritic cells were significantly increased in CF tissue compared with controls (*p* < 0.0001). This difference was seen in all compartments (airways: *p* = 0.0013, parenchyma: *p* < 0.0001 and perivascular: *p* = 0.012, versus control) (Table 2, Fig. 1). Likewise, langerin-positive (resident epithelial) dendritic cells (CD207) were significantly increased in CF tissue (*p* < 0.0001) with similar levels of significance for all compartments (airways: *p* < 0.0001, parenchyma: *p* < 0.0001 and perivascular: *p* = 0.0001, versus control) (Table 2, Fig. 1). When compared within CF lung tissue, both types of myeloid dendritic cells occurred more around airways compared to parenchyma (CD1a: *p* < 0.05, CD207: *p* < 0.01) or perivascular (CD1a: *p* < 0.01, CD207: *p* < 0.001). In control tissue, a similar observation was made for CD207 dendritic cells (airways compared to parenchyma and perivascular: both *p* < 0.001), while the increased presence of CD1a dendritic cells around the airways was only significant compared to parenchyma (*p* < 0.001), but not to the perivascular compartment (Additional file 1: Table S4).

Macrophage counts were significantly increased in CF tissue compared to controls (*p* = 0.0021) due to increased numbers found around the CF airways (*p* = 0.0049) and in the parenchyma (*p* = 0.011) (Table 2, Fig. 1). Neither CF nor controls showed any differences when compartmental distribution was evaluated (Additional file 1: Table S4).

### Lymphoid cell and follicle quantification and localization in end-stage CF lung tissue

**Fig. 2:**
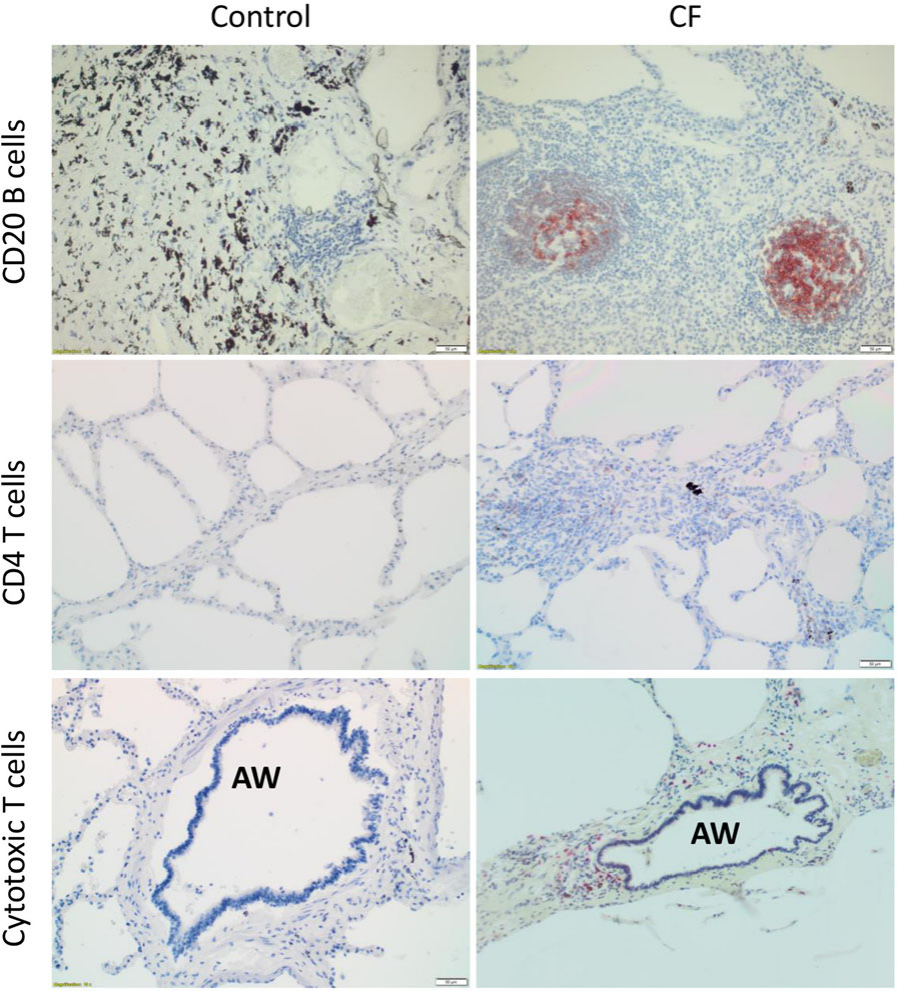
Histological sections of formalin-fixed paraffin-embedded human lung tissue of control subjects and CF patients. Sections were stained for B cells (CD20), CD4 T cells and cytotoxic T cells (CD8). Scale bar = 50 μm. Abbreviation: AW = airway

Both cytotoxic T cell (CD8) and CD4 T cell counts were significantly increased in CF tissue compared to controls (*p* < 0.001 and *p* = 0.007, respectively), whereas the slightly increased B cell counts in CF showed only a trend toward significance (*p* = 0.091 versus control) (Table 3, Fig. 2).

**Table 3:** Quantification of total lymphoid cell types in controls and CF patients, corrected for area unit

Lymphoid cell and follicle quantification (expressed as cells or follicles/mm^2^ area)			
	*Control subjects*	*CF patients*	*Mann-Whitney U test*
*CD4 T cells*	3.8 (0.6–7.6)	9.0 (2.9–21.7)	0.007**
*Cytotoxic T cells (CD8)*	1.5 (0.2–11.4)	55.8 (23.7–71.5)	<0.0001***
*B cells (CD20)*	0.0 (0.0–0.9)	0.3 (0.1–0.8)	0.091
*Lymphoid follicles*	0.0 (0.0–0.009)	0.056 (0.022–0.082)	<0.0001***
*Airway*	0.0 (0.0–0.003)	0.014 (0.006–0.029)	<0.0001***
*Parenchyma*	0.0 (0.0–0.003)	0.011 (0.006–0.039)	<0.0001***
*Perivascular*	0.0 (0.0–0.003)	0.011 (0.004–0.023)	0.0002***

During follicle quantification, localization was taken into consideration. The *p*-values in the right-hand column are the result of Mann-Whitney U testing. Significant differences between CF patients and control subjects are indicated with *, with * = *p* < 0.05, ** = *p* < 0.01 and *** = *p* < 0.001

data < 0.05 are captured in bold

**Fig. 3:**
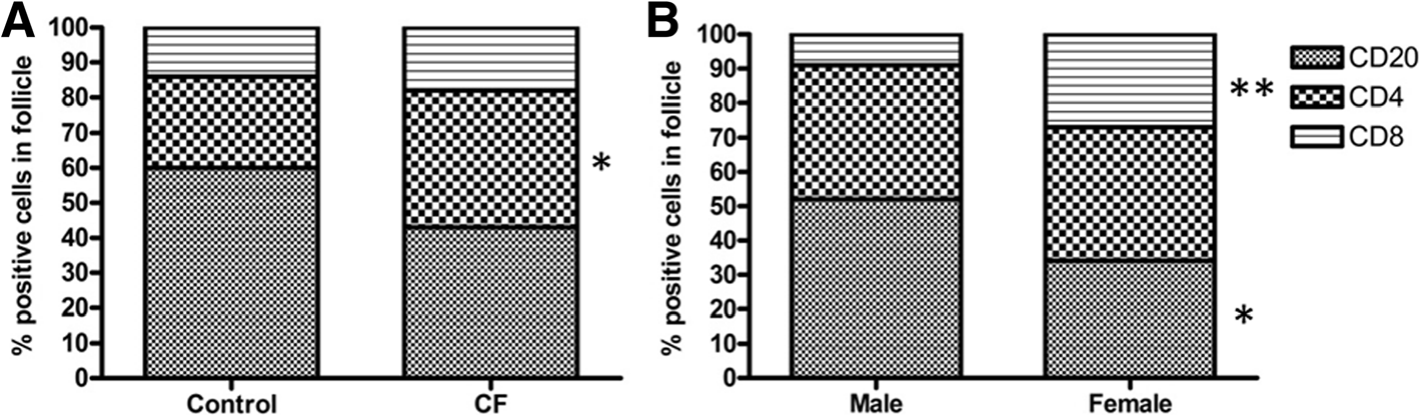
Composition of the lymphoid follicles found in the lung tissue. Proportions of CD20 B cells, CD4 T cells and CD8 T cells are expressed as percentage positive cells on the total amount of cells in the follicle. Panel (**a**) shows the difference in distribution of the follicular lymphoid cells between control subjects and CF patients, panel (**b**) concerns the distribution of the follicular lymphoid cells in CF males and females. Significant differences are indicated with *, with * = *p* < 0.05 and ** = *p* < 0.01

Lymphoid follicles (representative image: Additional file 1: Figure S2) were more abundant in CF tissue (*p* < 0.0001), which was observed in all three compartments (airways: *p* < 0.0001, parenchyma: *p* < 0.0001 and perivascular: *p* = 0.0002, versus control) (Table 3, Fig. 2). The size of the follicles did not differ between CF and control patients (data not shown) but the composition of the follicles did. In lymphoid follicles present in control tissue, CD20 B cells were more abundant than CD4 and CD8 T cells, resulting in a 60%–26%–14% distribution respectively. In CF tissue, the proportion of CD4 T cells was significantly increased (*p* = 0.020 versus control) giving a 43%–39%-18% (CD20, CD4 & CD8, respectively) distribution (Fig. 3a).

### Myeloid and lymphoid cell quantification in the male and female CF subpopulation

Table 4 shows the results after subdividing the CF patients in a male and female subpopulation. In control patients there was no significant difference between males and females in the number of myeloid cells, lymphoid cells or follicles present (data not shown).

**Table 4:** Quantification of myeloid and lymphoid cells in controls and CF, with male and female subdivision

Myeloid and lymphoid cell type quantification in the male and female CF patient subpopulation compared to control subjects				
	*Control subjects*	*Male CF patients*	*Female CF patients*	*ANOVA*
*Neutrophils (MPO)*	17.4 (7.1–30.5)	30.1 (25.4–45.5)	36.3 (24.0–51.8)*	**0.0009**
*Eosinophils (EG-2)*	1.0 (0.4–2.9)	0.3 (0.1–2.3)	4.7 (1.8–9.6)^§§^	**0.004**
*Mast cells (tryptase)*	10.5 (8.8–14.4)	12.5 (7.9–16.5)	23.0 (18.7–32.3)***^, §§^	**0.0005**
*Dendritic cells (CD1a)*	0.8 (0.4–1.5)	2.2 (1.5–3.9)*	3.6 (2.7–6.6)***	**<0.0001**
*Dendritic cells (CD207)*	1.0 (0.3–1.8)	4.3 (3.3–8.0)***	5.2 (2.8–14.3)***	**<0.0001**
*Macrophages (CD163)*	18.7 (10.3–25.1)	33.5 (23.2–43.2)**	26.9 (16.7–40.5)	**0.0058**
*CD4 T cells*	3.8 (0.6–7.6)	2.9 (1.2–7.1)	21.7 (10.8–29.8)***^, §§^	**<0.0001**
*Cytotoxic T cells (CD8)*	1.5 (0.2–11.4)	23.7 (12.7–58.6)*	71.5 (48.1–115.0)***	**<0.0001**
*B cells (CD20)*	0.0 (0.0–0.9)	0.5 (0.0–1.3)	0.3 (0.1–0.6)	0.21
*Lymphoid follicles*	0.0 (0.0–0.009)	0.04 (0.01–0.1)***	0.065 (0.028–0.071)***	**<0.0001**

Quantification of total myeloid and lymphoid cell counts in control subjects and CF patients, with in the latter group a subdivision in the male and female subpopulation. Myeloid cell types are expressed as cells/HPF, whereas the lymphoid cell types or follicles are expressed as cells or follicles/mm^2^ area. The *p*-values in the right-hand column are the result of Kruskal-Wallis 1-way ANOVA testing. Significant differences with control subjects are indicated with *, and significant differences with male CF patients are indicated with ^§^, with * = *p* < 0.05, ** = *p* < 0.01 and *** = *p* < 0.001. These values are the results of Dunn’s post hoc testing

data < 0.05 are captured in bold

Concerning the myeloid cells, both eosinophils and mast cells were significantly more abundant in female versus male CF patients (both *p* < 0.01). As for the lymphoid cells, CD4 T cell counts were increased in the female CF patients (*p* < 0.01). When comparing each gender to controls, the number of neutrophils, mast cells and CD4 T cells was significantly higher only in the female CF population (*p* < 0.05, *p* < 0.001 and *p* < 0.001 respectively). Macrophages on the other hand were only significantly increased in the male CF population (*p* < 0.01). CD20 B cells were equally present in male and female CF patients (*p* = 0.21). Organization in lymphoid tissue (number, size and distribution) was not different between male and female CF patients (Table 4). However, there was a significant difference in lymphoid follicle composition in female CF patients as B cells were less abundant (*p* = 0.043), whereas CD8 T cells were more abundant (*p* = 0.0029) than in male CF patients, resulting in a CD20-CD4-CD8 distribution of 52%–39%–9% respectively in males, and 34%–39%–27% respectively in females (Fig. 3b).

### Correlation with markers of inflammation

There were no correlations between the myeloid or lymphoid cell counts listed above, and any of the clinical inflammatory parameters (blood eosinophils, CRP, total IgE or IgG) measured in the peripheral blood of the CF patients (last available result before transplant procedure).

## Discussion

The present study demonstrated a significant increase of neutrophils, mast cells, CD1a and CD207 dendritic cells, macrophages, CD8 and CD4 T cells in CF lung tissue. Concomitantly, we found a disproportionate increase of neutrophils around the CF airways. The number of single B cells was not increased in the CF lung tissue. The number, but not the size of lymphoid follicles in CF lungs was increased and there was a skewed B cell/T cell composition. We also found some gender-specific changes in our CF population. Eosinophil, mast cell and CD4 T cell counts were specifically increased in lung tissue of female CF patients. Lastly, lymphoid follicles had less B cells and more CD8 T cells in females.

In this study, we have shown that the inflammatory signal in end stage CF lung disease is diverse and involves an increased abundance of both myeloid and lymphoid cells. As more and more players involved in CF lung inflammation are being identified, it becomes increasingly clear how diverse and disturbed the inflammatory pattern really is. This may be the consequence of the presence of mutated CFTR channels on various inflammatory cells. The absence of functional CFTR leads to impaired bacterial killing in murine and human macrophages and a deregulated release of pro-inflammatory cytokines by macrophages during the innate immune response [7]. As the CF lung is liable to infection by a myriad of pathogens, this may also promote the diversity of the immune response. By demonstrating an increase in T cells and lymphoid follicles in CF, we have confirmed the involvement of the cellular adaptive immune response in CF lung disease. A role for T cells in the pathophysiology of CF has already been suggested as CFTR present on circulating T cells participates in immune cell signalling. This CFTR-mediated signalling is disturbed in CF [7].

A striking finding was the large amount of lymphoid follicles found in all compartments of the CF lung. The presence of follicles is well-established in other chronic inflammatory diseases such as COPD [17]. B cell follicles are the result of lymphoid neogenesis leading to the development of tertiary lymphoid organs in tissues that are under a chronic and constant influence of certain triggers [17]. In end-stage CF, it seems logic that this trigger has an infectious nature as the lungs have endured years of microbial assaults and colonization with different pathogens. Due to the nature of this disease, these pathogens become very difficult to eradicate which results in a sustained immune response with infiltration into the lung parenchyma of macrophages, dendritic cells, T cells and B cells. These cells frequently organize themselves at an anatomical and functional level into tertiary lymphoid organs [17]. However, as CF is characterized by airway destruction, the B cell follicles could also reflect an adaptive immune response against degradation products of the extracellular matrix, becoming so-called neo-self antigens, and may in fact represent an autoimmune process that occurs after breaking the immune tolerance. In order to determine the aetiology of the lymphoid follicles found in CF, future research might focus on lung tissue derived from non-CF bronchiectasis patients who are often colonized with *P.aeruginosa, S.aureus and H.influenza* as well*.* If follicles are equally numerous in this disease, their development might have an infectious nature. In COPD, lymphoid follicles were described as aggregates of B cells that are surrounded by lower numbers of predominantly CD4 and to lesser extent CD8 T cells [17, 18]. However, in CF we observed a composition shift of the lymphoid follicles from B cell to T-cell predominance, suggesting that the cellular adaptive immune response is specifically affected in CF.

We also found that the increased inflammation in end-stage CF lung disease appeared to be gender-specific. Given the lack of apparent differences in patient characteristics between male and female CF patients in our population which may account for the observed female predominance in airway inflammation, there is some room for speculation. It has been shown that oestrogen has a complex immunomodulatory effect on inflammation and mucoid *P. aeruginosa* density [19]. Human CF patients whose lungs are actively infected with *P. aeruginosa* have elevated sputum levels of IL-23 and IL-17 which decrease dramatically following antibiotic treatment [20, 21]. Experiments with adult male mice demonstrated a critical role for IL-23 (and the Th17 products it induces) in the pathogenesis of murine lung inflammation upon infection with *P.* aeruginosa. Administration of the exogenous female sex hormone 17β-estradiol (circulating oestrogen) to male CF knock-out mice resulted in a more pronounced inflammatory reaction to infection with *P.* aeruginosa, which was reflected by increased lung tissue mRNA levels of IL-23 and IL-17 [22]. Sex hormones also modulate autoimmunity. Autoimmune diseases are a class of illnesses associated with increased Th17 activity and showing distinct gender-based differences in prevalence [19]. The gender-based difference in inflammation in our CF population may be the result of overstimulation of Th17 activity by oestrogens similar to what is seen in autoimmune diseases. Oestrogen can also cause a reduced production of IL-8 in CF bronchial epithelium through inhibition of NF-κB and IL-8 gene expression. This may result in a hyporesponsive innate immune response in the CF lung during times of high oestrogen exposure and predispose to infection and bacterial colonization [23].

The distinct inflammatory signature in females could have clinical implications. As there is a marked eosinophilic component, a more liberal policy with oral steroids (both during and independent of exacerbations) might be advocated in females. Future clinical trials with anti-inflammatory therapies using monoclonal antibodies may also want to analyse the effect in female CF patients separately.

Although the comprehensive pathological analysis of end-stage CF lung tissue provided a valuable insight in the inflammatory diversity of this disease, our study has some limitations. The origin of our samples only allowed us to study end-stage disease. To visualize disease progression, earlier stages of the disease will need to be studied in a similar way. The study of Regamey *et al.* who used endobronchial biopsies of children with a median age of 7.3 years to investigate the inflammatory pattern in the bronchial mucosa, already suggested that the infiltrate predominantly consists of macrophages and lymphocytes [24]. Although we did not specifically study the airway compartment when quantifying the lymphoid cells, it is clear that also in our study the CD4 and CD8 T cells outnumber the B cells, both as single dispersed cells as in the follicles, where in addition a composition shift to a larger proportion of CD4 T cells in CF compared to controls was noted. However, it seems that the lymphocytic infiltrate in the subepithelial tissue of children with CF has a larger portion of B cells (18% in CF compared to 13% in controls) which may indicate that the humoral adaptive response plays a bigger role in the earlier stages of CF lung disease. Moreover, Regamey *et al.* only found a limited number of neutrophils infiltrating the bronchial mucosa. The numerous presence of neutrophils around the airways in our study suggests that they infiltrate the tissue surrounding the airways in later stages of the disease, eventually leading to tissue destruction. A second limitation is that the number of CF and control tissue included is limited. As a result, we cannot exclude that the observed differences between male and female patients may be partly due to the small sample size. Thirdly, due to the large surface area of the biopsies and the fragile nature of CF lung tissue, we chose to cut nine μm thick sections. This could however confound our results in two ways: it may increase all cell type counts in CF tissue compared with controls, and it may also increase the number of large cell types like macrophages and neutrophils compared with small cells. Therefore, we described our findings in a qualitative way, not elaborating on the extent of the quantitative difference between CF and controls. Moreover, we have not compared cell types to each other but only looked at the differences between groups. A last limitation of our study is the age discrepancy between CF patients and control subjects, due to the fact that transplanted CF patients at our centre are inevitably younger than patients undergoing resection of bronchial carcinoma or in whom lung tissue was obtained after autopsy.

## Conclusion

By quantifying and localizing distinct myeloid and lymphoid subsets in CF and control lung tissue, the present study clearly showed a diverse inflammatory pattern in end-stage CF lung disease with an increased presence of neutrophils, mast cells, CD1a and CD207 dendritic cells, macrophages, CD8 and CD4 T cells. We also found a large amount of lymphoid follicles showing a composition shift from B cell to T cell predominance. Together with the elevated presence of single T cells and macrophages in CF tissue, this may suggest a role for the cellular adaptive immune system in the pathophysiology of CF. Furthermore, considering the specific increase of eosinophils, mast cells and CD4 T cell counts in the lung tissue of female CF patients, inflammation in the CF lung seemed to be gender-specific in our population. This may be the result of the complex immunomodulatory effects of oestrogen on inflammation.

## Additional file

Additional file 1:
**Table S1.** Detailed overview of the used primary and secondary antibodies, and chromogens. Superscript numbers (1–5) above the catalogue number indicate which primary antibody is combined with which secondary antibody and chromogen. Abbreviation: RTU = ready to use. **Table S2.** Inter- and intra-observer variability in the counting of the myeloid and lymphoid cells, expressed by means of the Spearman’s rank correlation coefficient. Significant correlations are indicated with *, with *** = *p* < 0.001. **Table S3.** Overview of the number of analysed follicles in each compartment for every included tissue block. CF11-20 represents the female CF patients. **Table S4.** Quantification of the myeloid cell types and lymphoid follicles according to localization (subdivided in the three compartments: airways, parenchyma and perivascular). The *p*-values in the right-hand column are the result of Kruskal-Wallis 1-way ANOVA testing. Significant differences with airways are indicated with *, with * = *p* < 0.05, ** = *p* < 0.01 and *** = *p* < 0.001. These values are the results of Dunn’s post hoc testing. **Figure S1.** Histological section of formalin-fixed paraffin-embedded CF lung tissue. Section was stained for mast cells (tryptase). Representative image showing an airway surrounded by circular fibrosis suggestive of constrictive bronchiolitis. The bronchiole is accompanied by its blood vessel. Abbreviations: AW = airway, BV = blood vessel. Scale bar = 100 μm. **Figure S2.** Serial histological sections of formalin-fixed paraffin-embedded CF lung tissue. Both images show the same lymphoid follicles located in the proximity of an airway. Panel A shows a CD20 staining of all B cells lying organised in germinal centres. These are surrounded by a paracortex staining positively for CD3 T cells (panel B). High endothelial venules (green arrowhead) allowing extravasation of naïve B and T cells into the lymphoid follicle are located in the T cell area. AW = airway. Scale bar = 50 μm. (DOCX 19517 kb)

## Abbreviations

AB: Antibiotic
CF: Cystic fibrosis
CFTR: Cystic fibrosis transmembrane conductance regulator gene
CRP: C-reactive protein
FEV_1_: Forced expiratory volume in 1 s
HPF: High power field
I.V.: Intravenous
Ig: Immunoglobulin
IL: Interleukin
LTx: Lung transplantation
NF-κB: Nuclear factor kappa-light-chain-enhancer of activated B cells
WBC: White blood cells
