# Supplementary material for: End-stage cystic fibrosis lung disease is characterised by a diverse inflammatory pattern: an immunohistochemical analysis
Source: Respir Res. 2017 Jan 10;18:10. doi: 10.1186/s12931-016-0489-2 (PMC5223576; doi:10.1186/s12931-016-0489-2)
Supplement: Additional file 1: — Table S1. Detailed overview of the used primary and secondary antibodies, and chromogens. Superscript numbers (1–5) above the catalogue number indicate which primary antibody is combined with which secondary antibody and chromogen. Abbreviation: RTU = ready to use. Table S2. Inter- and intra-observer variability in the counting of the myeloid and lymphoid cells, expressed by means of the Spearman’s rank correlation coefficient. Significant correlations are indicated with *, with *** = p < 0.001. Table S3. Overview of the number of analysed follicles in each compartment for every included tissue block. CF11-20 represents the female CF patients. Table S4. Quantification of the myeloid cell types and lymphoid follicles according to localization (subdivided in the three compartments: airways, parenchyma and perivascular). The p-values in the right-hand column are the result of Kruskal-Wallis 1-way ANOVA testing. Significant differences with airways are indicated with *, with * = p < 0.05, ** = p < 0.01 and *** = p < 0.001. These values are the results of Dunn’s post hoc testing. Figure S1. Histological section of formalin-fixed paraffin-embedded CF lung tissue. Section was stained for mast cells (tryptase). Representative image showing an airway surrounded by circular fibrosis suggestive of constrictive bronchiolitis. The bronchiole is accompanied by its blood vessel. Abbreviations: AW = airway, BV = blood vessel. Scale bar = 100 μm. Figure S2. Serial histological sections of formalin-fixed paraffin-embedded CF lung tissue. Both images show the same lymphoid follicles located in the proximity of an airway. Panel A shows a CD20 staining of all B cells lying organised in germinal centres. These are surrounded by a paracortex staining positively for CD3 T cells (panel B). High endothelial venules (green arrowhead) allowing extravasation of naïve B and T cells into the lymphoid follicle are located in the T cell area. AW = airway. Scale bar = 50 μm. (DOCX 19517 kb) [file 12931_2016_489_MOESM1_ESM.docx]

***Online supplementary material***

**Materials and methods**

**Study material**

Patient data were collected via the electronic patient files from the Leuven University Hospitals or from the referring centre if insufficient data were present in the own data base. Characteristics collected include sex, age, mutation type, presence of nasal polyposis, forced expiratory volume in 1 second (FEV_1_), days on regular transplant or high urgency waiting list, C-reactive protein (CRP) level, percentage blood eosinophilia, total IgE level, *Pseudomonas aeruginosa* colonization status, presence of fungi in the explant lung material (fungal species encountered were *Aspergillus fumigatus, Penicillium species and Scedosporium apiospermum*, cultured either directly from explant lung tissue or from explant lung broncho-alveolar lavage fluid), use of inhaled or oral corticosteroids and time to last pre-LTx I.V. antibiotic (AB) therapy.

**Immunohistochemistry**

Antigen retrieval with Tris-EDTA (pH9) was required for CD4 and CD8 and with sodium citrate (10 mM, pH6) for MPO, tryptase and CD163. Sections were first incubated with a mouse anti-human monoclonal antibody and subsequently with an appropriate secondary antibody linked with biotin-avidin/streptavidin peroxidase (Vector, Burlingame, CA, USA), horse radish peroxidase (HRP) (Dako) or BOND polymer refine red detection (Leica, Newcastle, UK). Supplementary table 1 gives an overview of all used primary and secondary antibodies together with an appropriate chromogen. When secondary antibodies linked with HRP were used, endogenous peroxidase activity was blocked by incubating with peroxidase blocking solution (Dako) for five minutes. Next, the staining was visualized using AEC chromogen (Dako), new fuchsine (Dako) or fast red (Leica). Eventually, all sections were counterstained with Mayer’s hematoxylin and mounted with Vecta Mount (Vector).

**Image analysis**

For each patient and control subject, every lymphoid follicle encountered was photographed. This was done separately for the CD20 B cell, CD4 T cell and CD8 T cell staining. Then, the follicles were subdivided according to their location: in the parenchyma, perivascular or in the proximity of an airway. The number of analyzed follicles in each compartment is shown in supplementary table 3. CF11-20 represents the female CF patients. These numbers were then divided by the surface area of the section to obtain the number of follicles/mm² area. Then, using the photographs, the size of the follicles was determined. Furthermore, these photographs allowed us to make an estimate of the percentage positive cells within the follicles. For each staining (CD20-CD4-CD8), we allocated a percentage (in steps of 10%) of positivity to each individual follicle (therefore, each follicle was included in the analysis) which we used to study differences in composition.

| **Primary antibodies** | | | | |
| --- | --- | --- | --- | --- |
| *Antigen* | *Catalogue number* | *Vendor* | *Clone number* | *Dilution* |
| CD1a | M3571^1^ | Dako, Heverlee, Belgium | 010 | 1:25 |
| CD4 | M7310^2^ | Dako, Heverlee, Belgium | 4B12 | 1:50 |
| CD8 | M7103^2^ | Dako, Heverlee, Belgium | C8/144B | 1:50 |
| CD20 | M0755^2^ | Dako, Heverlee, Belgium | L26 | 1:250 |
| CD163 | NCL-CD163^3^ | Leica, Newcastle, UK | 10D6 | 1:200 |
| CD207 | IM3449^4^ | Immunotech, Marseille, France | DCGM4 | 1:100 |
| EG-2 | EG2^5^ | Diagnostics Development | EG2 | 1:100 |
| MPO | A0398^3^ | Dako, Heverlee, Belgium | Polyclonal | 1:2000 |
| Tryptase | Ab81703^2^ | Dako, Heverlee, Belgium | AA1 | 1:100 |

| **Secondary antibodies** | | | |
| --- | --- | --- | --- |
| *Type* | *Catalogue number* | *Vendor* | *Dilution* |
| Poly-AP goat anti-mouse IgG (AP) | DPVM-55AP^1^ | Klinipath, Duiven, The Netherlands | RTU |
| Goat anti rabbit/mouse (HRP) | K4063^2^ | Dako, Heverlee, Belgium | RTU |
| Rabbit anti-mouse IgG (AP) | DS9390^3^ | Leica, Newcastle, UK | RTU |
| Poly-AP IgG (AP) | DS9390^3^ | Leica, Newcastle, UK | RTU |
| Poly-AP anti-rat IgG (AP) | RT518G^4^ | Biocare Medical | RTU |
| Goat anti-mouse (biotin) | BA-9200^5^ | Vector, CA, USA | 1:50 |
| Streptavidin peroxidase (HRP) | PK-6100^5^ | Vector, CA, USA | RTU |

| **Chromogens** | | | | |
| --- | --- | --- | --- | --- |
| *Name* | *Catalogue number* | *Vendor* | *Colour* | *Dilution* |
| AEC (HRP) | K3462^2, 5^ | Dako, Heverlee, Belgium | Red | RTU |
| Fast red (AP) | DS9390^3^ | Leica, Newcastle, UK | Pink | RTU |
| New Fuchsin (AP) | KO625^1, 4^ | Dako, Heverlee, Belgium | Pink | RTU |

**Table 1:** Detailed overview of the used primary and secondary antibodies, and chromogens. Superscript numbers ^(1, 2, 3, 4, 5)^ above the catalogue number indicate which primary antibody is combined with which secondary antibody and chromogen. Abbreviation: RTU = ready to use.

| **Inter- and intra-observer correlations** | | | | |
| --- | --- | --- | --- | --- |
|  | *Inter-observer variability* | | *Intra-observer variability* | |
|  | *Spearman’s rank*  *correlation coefficient* | p-value | *Spearman’s rank*  *correlation coefficient* | p-value |
| *Neutrophils (MPO)* | 0.92 | **<0.0001***** | 0.94 | **<0.0001***** |
| *Eosinophils (EG-2)* | 0.86 | **<0.0001***** | 0.94 | **<0.0001***** |
| *Mast cells (tryptase)* | 0.9 | **<0.0001***** | 0.89 | **<0.0001***** |
| *Dendritic cells (CD1a)* | 0.91 | **<0.0001***** | 0.87 | **<0.0001***** |
| *Dendritic cells (CD207)* | 0.90 | **<0.0001***** | 0.94 | **<0.0001***** |
| *Macrophages (CD163)* | 0.88 | **<0.0001***** | 0.89 | **<0.0001***** |

**Table 2:** Inter- and intra-observer variability in the counting of the myeloid and lymphoid cells, expressed by means of the Spearman’s rank correlation coefficient. Significant correlations are indicated with *, with ***=p<0.001.

**Table 3:** Overview of the number of analysed follicles in each compartment for every included tissue block. CF11-20 represents the female CF patients.

| **Lymphoid follicle counts** | | | | | | | | |
| --- | --- | --- | --- | --- | --- | --- | --- | --- |
|  | *In the parenchyma* | | *In the proximity of an airway* | | | *Perivascular* | | *Total* |
| *CF1* | 22 | | 0 | | | 13 | | 35 |
| *CF2* | 1 | | 0 | | | 0 | | 1 |
| *CF3* | 3 | | 3 | | | 0 | | 6 |
| *CF4* | 0 | | 3 | | | 2 | | 5 |
| *CF5* | 9 | | 16 | | | 0 | | 25 |
| *CF6* | 2 | | 0 | | | 0 | | 2 |
| *CF7* | 5 | | 3 | | | 4 | | 12 |
| *CF8* | 23 | | 16 | | | 2 | | 41 |
| *CF9* | 15 | | 2 | | | 13 | | 30 |
| *CF10* | 14 | | 8 | | | 7 | | 29 |
| *CF11* | 4 | | 15 | | | 5 | | 24 |
| *CF12* | 9 | | 4 | | | 4 | | 17 |
| *CF13* | 10 | | 6 | | | 2 | | 18 |
| *CF14* | 0 | | 7 | | | 1 | | 8 |
| *CF15* | 4 | | 22 | | | 6 | | 32 |
| *CF16* | 4 | | 11 | | | 13 | | 28 |
| *CF17* | 6 | | 3 | | | 7 | | 16 |
| *CF18* | 3 | | 3 | | | 2 | | 8 |
| *CF19* | 1 | | 5 | | | 9 | | 15 |
| *CF20* | 16 | | 17 | | | 12 | | 45 |
| *Control 1* | 0 | | 0 | | | 0 | | 0 |
| *Control 2* | 0 | | 0 | | | 0 | | 0 |
| *Control 3* | 0 | | 0 | | | 0 | | 0 |
| *Control 4* | 0 | | 0 | | | 2 | | 2 |
| *Control 5* | 0 | | 3 | | | 1 | | 4 |
| *Control 6* | 0 | | 0 | | | 0 | | 0 |
| *Control 7* | 0 | | 0 | | | 0 | | 0 |
| *Control 8* | 1 | | 1 | | | 0 | | 2 |
| *Control 9* | 0 | | 0 | | | 0 | | 0 |
| *Control 10* | 1 | | 1 | | | 2 | | 4 |
| *Control 11* | 0 | | 0 | | | 0 | | 0 |
| *Control 12* | 1 | | 0 | | | 0 | | 1 |
| *Control 13* | 2 | | 1 | | | 1 | | 4 |
| *Control 14* | 0 | | 0 | | | 0 | | 0 |
| *Control 15* | 1 | | 0 | | | 1 | | 2 |
| *Control 16* | 0 | | 0 | | | 0 | | 0 |
| *Control 17* | 0 | | 0 | | | 0 | | 0 |
| *Control 18* | 0 | | 0 | | | 0 | | 0 |
| *Control 19* | 0 | | 1 | | | 0 | | 1 |
| *Control 20* | 0 | | 2 | | | 2 | | 4 |
| *Control 21* | 1 | | 0 | | | 0 | | 1 |
| *Control 22* | 0 | | 0 | | | 0 | | 0 |
| **Localization of myeloid cells and lymphoid follicles in CF patients and control subjects (expressed as cells/HPF or follicles/mm² area)** | | | | | | | | |
|  | | *Airways* | | *Parenchyma* | *Perivascular* | | *p-value* | |
| *Neutrophils (MPO)*  *Control subjects*  *CF patients* | | 15.1 (6.3-28.7)  67.5 (43.5-81.9) | | 19.7 (7.1-40.6)  28.9 (15.3-36.6)** | 9.7 (3.5-19.4)  15.2 (9.2-18.9)*** | | 0.17  **<0.0001** | |
| *Eosinophils (EG-2)*  *Control subjects*  *CF patients* | | 1.7 (0.6-5.5)  2.0 (0.5-7.7) | | 1.1 (0.3-2.7)  1.1 (0.1-3.6) | 0.6 (0.1-1.7)  0.4 (0.0-2.0) | | 0.089  0.11 | |
| *Mast cells (tryptase)*  *Control subjects*  *CF patients* | | 18.8 (15.3-31.2)  25.2 (19.9-36.6) | | 7.8 (5.0-9.2)***  15.0 (8.8-21.3)* | 9.3 (6.9-11.1)***  12.5 (9.9-15.8)** | | **<0.0001**  **0.001** | |
| *Dendritic cells (CD1a)*  *Control subjects*  *CF patients* | | 2.3 (0.8-3.6)  4.7 (3.0-6.3) | | 0.2 (0.0-0.5)***  2.2 (1.0-3.8)* | 0.7 (0.1-2.0)  1.6 (0.9-3.5)** | | **0.0004**  **0.004** | |
| *Dendritic cells (CD207)*  *Control subjects*  *CF patients* | | 3.0 (1.6-4.7)  9.6 (7.6-13.2) | | 0.0 (0.0-0.2)***  2.7 (1.1-5.9)** | 0.1 (0.0-0.4)***  1.2 (0.5-4.0)*** | | **<0.0001**  **0.0002** | |
| *Macrophages (CD163)*  *Control subjects*  *CF patients* | | 15.5 (8.7-26.6)  33.8 (18.4-40.4) | | 21.3 (10.6-26.8)  35.2 (21.9-46.8) | 16.9 (11.0-21.0)  22.4 (14.1-31.6) | | 0.62  0.068 | |
| *Lymphoid follicles*  *Control subjects*  *CF patients* | | 0.00 (0.00-0.00)  0.01 (0.01-0.03) | | 0.00 (0.00-0.00)  0.01 (0.01-0.04) | 0.00 (0.00-0.00)  0.01 (0.00-0.02) | | 1.00  0.52 | |

**Table 4:** Quantification of the myeloid cell types and lymphoid follicles according to localization (subdivided in the three compartments: airways, parenchyma and perivascular). The p-values in the right-hand column are the result of Kruskal-Wallis 1-way ANOVA testing. Significant differences with airways are indicated with *, with *=p<0.05, **=p<0.01 and ***=p<0.001. These values are the results of Dunn’s post hoc testing.


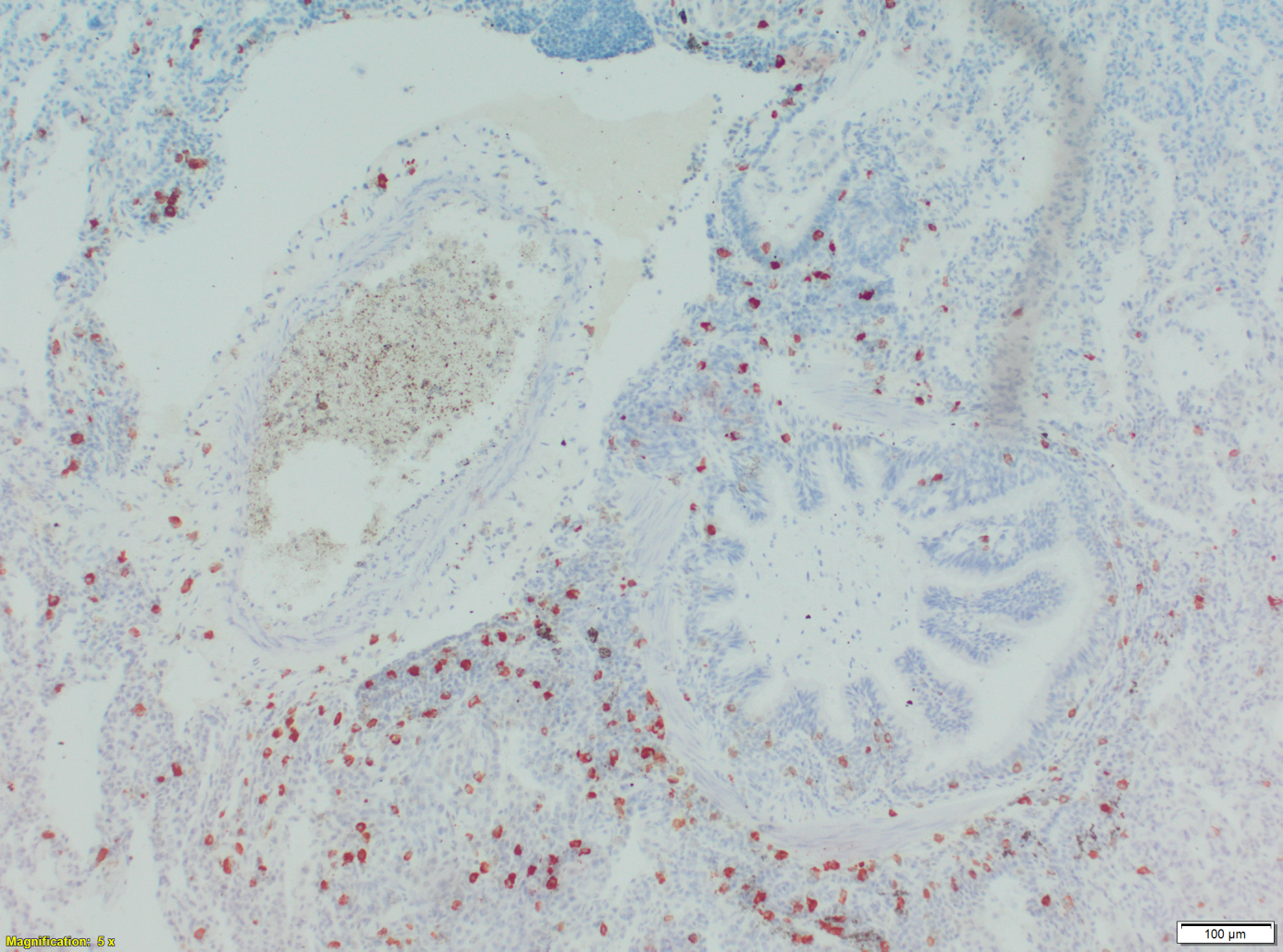


**BV**

**AW**

**Figure 1:** Histological section of formalin-fixed paraffin-embedded CF lung tissue. Section was stained for mast cells (tryptase). Representative image showing an airway surrounded by circular fibrosis suggestive of constrictive bronchiolitis. The bronchiole is accompanied by its blood vessel. Abbreviations: AW = airway, BV = blood vessel. Scale bar= 100 µm

**Figure 2:** Serial histological sections of formalin-fixed paraffin-embedded CF lung tissue. Both images show the same lymphoid follicles located in the proximity of an airway. Panel A shows a CD20 staining of all B cells lying organised in germinal centres. These are surrounded by a paracortex staining positively for CD3 T cells (panel B). High endothelial venules (green arrowhead) allowing extravasation of naïve B and T cells into the lymphoid follicle are located in the T cell area. AW = airway. Scale bar = 50 µm.

**CD20**


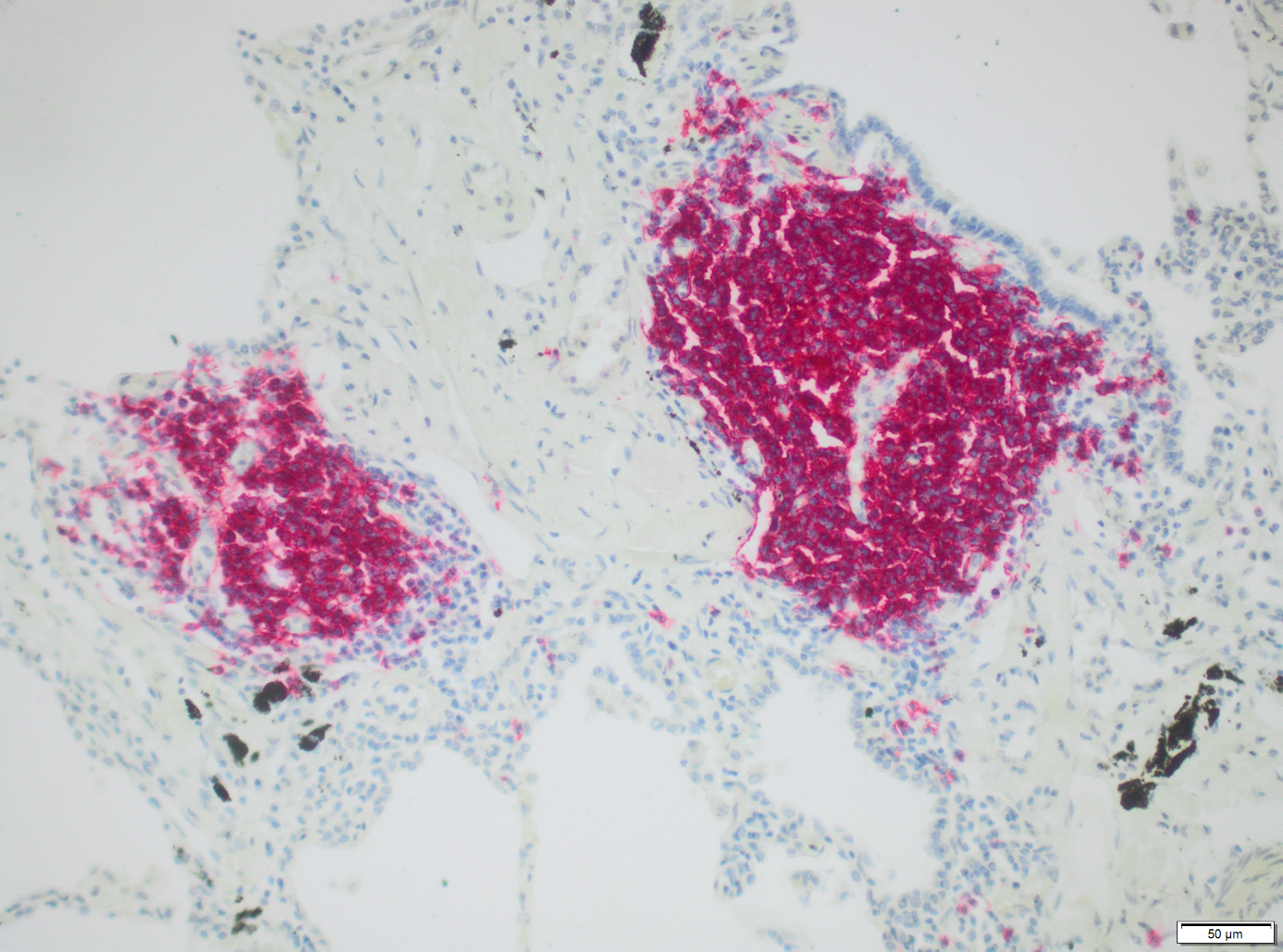

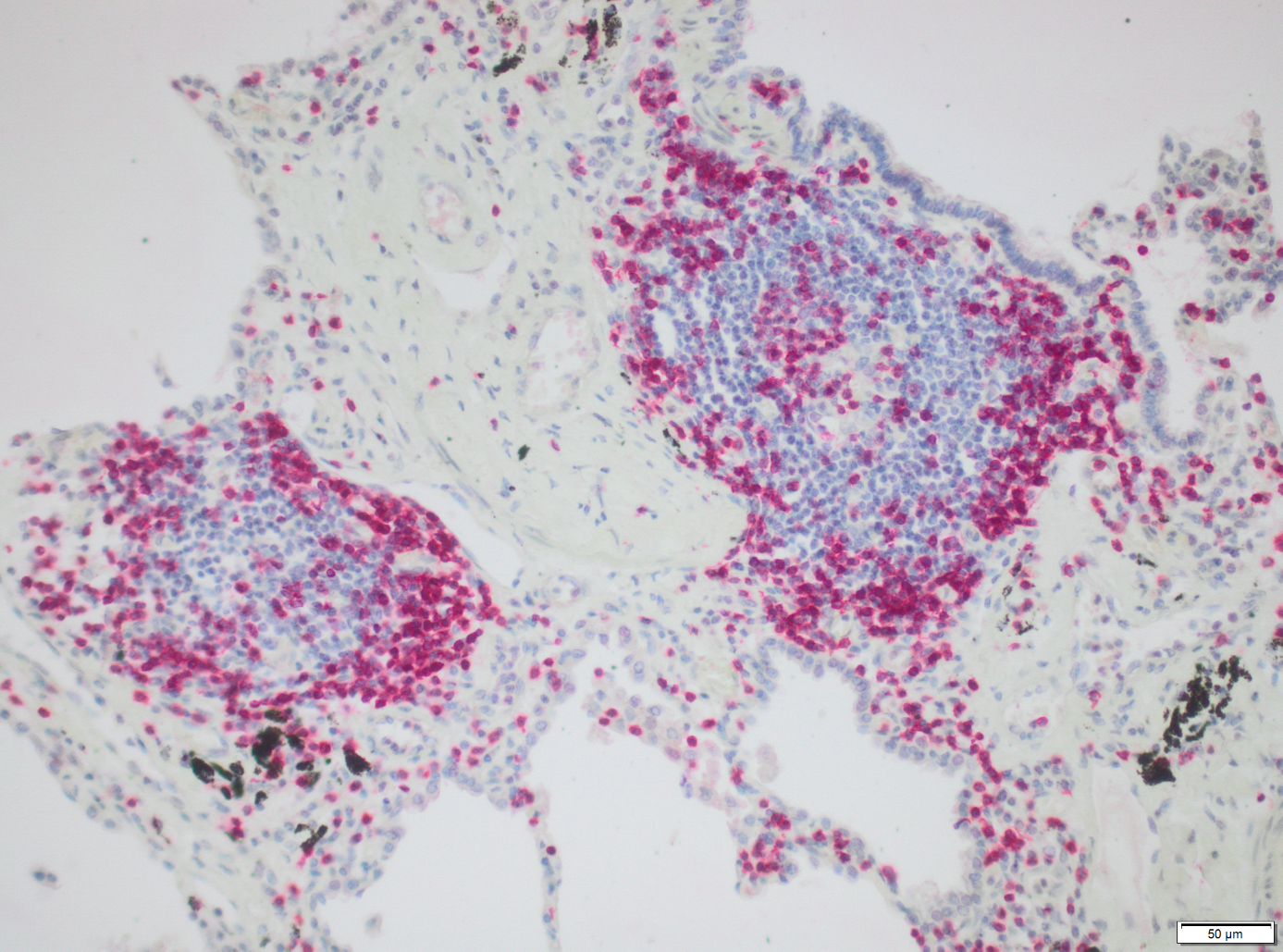


**A**

**B**

**AW**

**CD3**

**AW**
